# Process and Pitfalls of Sperm Cryopreservation

**DOI:** 10.3390/jcm6090089

**Published:** 2017-09-19

**Authors:** Hamoun Rozati, Thomas Handley, Channa Jayasena

**Affiliations:** Section of Investigative Medicine, Imperial College London, Hammersmith Hospital, Du Cane Road, London W12 0NN, UK; h.rozati@imperial.ac.uk (H.R.); thomas.handley15@imperial.ac.uk (T.H.)

**Keywords:** cancer, fertility, sperm, cryopreservation, ethics

## Abstract

Sperm cryopreservation has been utilized routinely for over 40 years to preserve fertility in men undergoing cancer therapy and allow conception for infertile couples. This article provides a concise and up-to-date review of the literature and covers the latest advances in sperm cryopreservation and its array of clinical indications. Over recent years, the scope of clinical indications used for sperm cryopreservation has expanded widely. Consequently, more patient groups are eligible for sperm freezing, requiring specialist resources and higher running costs. Although sperm cryopreservation prior to cancer therapy is readily available in many countries, referral rates by oncology specialists and levels of patient engagement with cryopreservation services are both reported as low. Furthermore, sperm banking continues to raise ethical issues such whether sperm donation should be anonymous and whether sperm can be utilized posthumously by the surviving partner without consent from the patient. This review focuses on the technological advances and ethical controversies in sperm cryopreservation, and how better understanding of these issues could lead to improved access to fertility preserving treatment for patients.

## 1. Introduction

Sperm cryopreservation (“sperm freezing”) has been used widely since the 1970s to treat couples with infertility. Sperm freezing is principally used to store sperm in patients undergoing cancer therapy and play a vital role in treating couples with infertility. However, several other clinical applications for sperm freezing have emerged in recent years. Furthermore, sperm banking remains an emotive subject raising ethical debates in society. This article aims to provide a concise and contemporary review summarizing the major clinical applications, challenges and future avenues for sperm freezing.

## 2. Technical Considerations for Sperm Freezing

The first human live birth using frozen sperm was reported in 1953 following short-term storage of semen in dry ice [1]. By 1963, liquid nitrogen was introduced as a method of long-term sperm cryopreservation, which allowed sperm freezing to become widespread in healthcare [2,3]. Sperm can be used successfully during fertility therapy even after 40 years of cryopreservation [4]. However, liquid nitrogen confers a risk of viral cross-contamination with other sperm samples in the same container [5]. Consequently, most modern sperm storage banks utilize nitrogen vapor, which theoretically confers a much lower risk of viral cross-contamination [6].

The simplest method of producing a semen sample for sperm banking is masturbation; however, other techniques are available if this is not possible. Disposal penile vibratory stimulation devices are noninvasive and simple [7]. Electro-ejaculation may be more required in cases associated with disruption of the ejaculatory reflex arc such as in spinal cord injuries [8], but requires a general anaesthetic. Surgical sperm retrieval techniques such as epidermal sperm aspiration [9] and testicular sperm extraction may be useful when a low sperm yield is anticipated, or when electro-ejaculation is not available [10].

Pre-freeze semen parameters have been shown to be an accurate predictor of post-thaw sperm motility and viability [11]. However, sperm undergo damage during the freezing and thawing process due to osmotic and oxidative stress, toxicity from the cryoprotectant and the formation of intracellular ice crystals [12]. For example, freeze-thawing causes a reduction in the number of normally functional sperm [13]. The process of cryopreservation must therefore be controlled to minimize the risks of damage to sperm. The most commonly used cryoprotectant is glycerol mixed with egg yolk, which reduces the incidence of osmotic stress within spermatozoa [14]. Research has been conducted into the addition of further cryoprotective agents to maintain the integrity of stored sperm, including zinc [15] and resveratrol and ascorbic acid [16]. Production of reactive oxygen species (ROS) may contribute to the deterioration of sperm function during cryopreservation [17]. The effects of ROS have been shown to be mitigated by the addition of antioxidants such as the TAT-peroxiredoxin-2 fusion protein [18]. The use of additives to cryopreserved sperm is an area that requires further investigation before becoming routine practice.

Numerous studies have investigated whether advanced sperm selection techniques to improve post-thaw semen quality, mainly focused on selection of sperm by hyaluronanic acid binding; however, a recent Cochrane review was unable to recommend their routine use [19]. Sperm washing separates spermatozoa from seminal plasma prior to freezing, and has been reported to improve post-thaw sperm motility [20]. The American Society for Reproductive Medicine considers sperm washing to be the standard of care in patients with HIV [21]. Sperm washing has been performed on sperm samples from patients with human immunodeficiency virus (HIV) by one UK center since 1999; no seroconversions have been reported during pregnancies arising from samples undergoing sperm washing to date [22]. A number of methods for sperm washing exist, the most common being semen centrifugation in a 45–90% colloidal silica density gradient to separate HIV-free progressively motile sperm from the infected seminal plasma and non-sperm cells such as leukocytes.

## 3. Sperm Banking for Cancer

### 3.1. Risk Factors for Infertility during Cancer

Malignancy is the major indication for sperm banking. Testicular cancer, Hodgkin’s lymphoma and leukemia confer higher risks of male infertility when compared with other tumor types [23]. Rates of recovery of spermatogenesis are highly variable and dependent on cancer type, treatment modality and underlying testicular function, but even highly gonadotoxic treatment for testicular cancer can see recovery of spermatogenesis in 50% of patients after two years [24]. For example, the Childhood Cancer Survivor study demonstrated that 46% of survivors of childhood cancer reported infertility when compared with 17.5% of their siblings [25]. The same study suggested that bleomycin treatment, a testicular radiation dose ≥4 Gy, an alkylating dose score ≥3 and surgical excision of any organ in the genital tract were significant risk factors for infertility [26]. Other risk factors associated with male fertility during cancer therapy are summarized in Table 1. Biological therapies are used increasingly to treat men with cancer. There is evidence that mammalian target of rapamycin (mTOR) inhibitors such as everolimus may impair gonadal function [27]. Case reports [28] and in vitro studies [29] suggest the tyrosine kinase inhibitor imatinib may impair spermatogenesis. The American Society of Clinical Oncology (ASCO) therefore recommend counselling young males with chronic myeloid leukemia that there is insufficient evidence to exclude that tyrosine kinase inhibitor therapy could affect their long-term fertility [30]. Further research is required to elucidate to what extent novel biological agents represent an indication for sperm freezing in men with cancer.

### 3.2. Barriers to Sperm Freezing during Cancer

A survey in the UK revealed that only 38% of oncology physicians provided patients with cancer with written information about fertility; contributory factors included having insufficient time to discuss sperm freezing, a lack of knowledge and poor perceived success of fertility preservation [59]. Furthermore, 48% of oncology physicians in the United States admitted discussing the subject of fertility preservation with less than a quarter of patients with cancer [60]. Certain patient groups may also require further support when engaging with sperm freezing services. In the USA, patients without a college degree or private healthcare insurance, and with children aged younger than 18 years were statistically less likely to engage with fertility preservation than other patients with cancer [61]. Clinicians may be particularity uncomfortable discussing regarding fertility preservation with adolescent patients with cancer [62]. However, a large-scale study in France of 4345 patients aged 11–20 years with cancer suggests that a semen sample can be produced in 93% of cases. It is noteworthy that an 81% success rate was observed in patients aged 11–14 years within this study [63]. Collectively, these data suggest that nonreferral of patients with cancer at risk of infertility is high, and particular challenges are faced when ensuring adolescents with cancer gain the most out of available fertility preservation services.

### 3.3. Testicular Toxicity in Cancer

There are currently no recommended approaches to protect spermatogenesis in patients undergoing chemotherapy [30]. Several studies have investigated whether hormonal suppression of testicular function could protect against germ cell loss. Pretreatment with a gonadotrophin-releasing hormone (GnRH) antagonist significantly increases testicular weight and tubule differentiation in monkeys undergoing testicular irradiation followed by spermatagonial stem cell transplantation [64]. However, historical studies to date have failed to observe any significant benefit of hormonal suppression of testicular function (using GnRH agonists, testosterone, androgenic progestogens or anti-androgens) on sperm function in men with cancer [65]. Despite this, spermatogenesis recovers in many patients following cancer therapy. The patient may therefore require guidance whether to retain their frozen and pretreatment sperm if concerns exist regarding the quality of their ejaculated sperm post-treatment. ASCO recommends counselling patients that sperm collected after starting treatment is at higher than normal risk of genetic damage [30]. However, it is also important to counsel patients that conceiving naturally (albeit using post-treatment sperm) could avoid the need for invasive and potentially expensive assisted reproductive technologies (ART). It is therefore important to consider what markers could help clinicians and patients decide how much sperm damage has been sustained following cancer therapy. Abnormal sperm morphology is statistically associated with lower pregnancy rates following IVF, but its diagnostic accuracy is poor (AUC 54%) [66]. Fluorescence in situ hybridization (FISH) analysis may be used to determine the proportion of sperm with aneuploidy [67]. Novel methods of assessing fertility in male patients with cancer include assessing sperm deoxyribonucleic acid (DNA) methylation patterns [68] and levels of sperm DNA fragmentation in thawed samples [69]. Anti-Müllerian hormone (AMH) has been demonstrated as a surrogate marker for testicular damage in mice studies [70]. Though showing promise, these are not currently used in routine practice.

## 4. Autologous and Donor Sperm Banking for Infertility

Sperm freezing is an established tool in the management of couples with infertility, and the scope of its clinical applications is widening rapidly. Autologous sperm freezing is commonly utilized to provide a backup sample for patients with severe oligospermia undergoing intracytoplasmic sperm injection (ICSI). Sperm retrieved during epididymal sperm aspiration or testicular biopsy may be frozen if the procedure cannot be performed synchronously with egg retrieval during ART. Sperm banking may also be provided by a sperm donor when no suitable sperm can be produced by the patient. Other indications for sperm donation may include preventing vertical transmission of an infectious or genetic condition, and fertility treatment for single women or women in same-sex relationships [14,71]. Men must undergo rigorous clinical assessment prior to sperm donation [72]. Serum blood tests are performed to exclude viral infections such as HIV, hepatitis B and C, syphilis, human T-lymphotropic virus (HTLV) and cytomegalovirus (CMV); recent outbreaks of viruses such as Ebola [31] and Zika [73] dictate that sperm bank policies must evolve constantly to ensure subgroups of sperm donors are screening appropriately for viral pathogens. Genetic testing should be performed in sperm donors if indicated by prescreening history and questionnaire, although the authors would not recommend its use routinely. Advanced age has been linked with an increasing number of defects within sperm genomes [74] and in embryos formed as a result of ART using donor sperm [75]. Accordingly, the American Society for Reproductive Medicine (ASRM) and Society for Assisted Reproductive Technology recommend sperm donors should ideally be aged younger than 40 years. Furthermore, the Human Fertilisation and Embryology Authority (HFEA) in the UK recommend an upper limit of 41 years in sperm donors [76]. However, a recent meta-analysis suggests that advancing paternal age is not associated negatively with pregnancy, miscarriage or live births in an oocyte-donation model [77]. In summary, sperm donation uses sperm which is either autologous or donor in origin to allow conception in couples who may be heterosexual, homosexual or transgender in identity.

## 5. Sperm Banking for Gender Reassignment

A recent meta-analysis suggested that the prevalence of transgender individuals is 4.6/100,000 in the populations studied [78]. Nearly three times as many individuals undergo male-to-female reassignment (“trans women”) when compared with female-to-male reassignment (“trans men”). Trans women are treated with estrogen therapy with or without bilateral orchiectomy, which would render them temporarily or permanently infertile. However, transgender individuals often desire and expect to conceive in the future with appropriate fertility treatment [79]. For this reason, the Endocrine Society recommends that fertility counselling should be offered to patients undergoing gender reassignment [80]. Furthermore, the ethics committee of the ASRM recommends offering the option of gamete freezing to all individuals undergoing gender reassignment [81]. However, further studies are required to explore how effectively sperm banking is delivered by health professionals and utilized for ART by patients. In conclusion, sperm banking is emerging as an important element of gender reassignment therapy; however, many clinicians have limited knowledge of this novel therapeutic pathway, and further work is needed to develop clinical pathways to treat individuals seeking treatment.

## 6. Sperm Banking for Other Indications

Sperm retrieval and freezing may be required in patients for whom ejaculation is not possible. Spinal cord injury (SCI) causes erectile dysfunction, anejaculation and reduced semen quality [82]. Abdominopelvic surgery including retroperitoneal lymph node dissection (RPLND), aorto-iliac reconstruction and colorectal excision may also cause anejaculation [58,83]. Medical causes of ejaculatory failure includes multiple sclerosis, with an ejaculatory dysfunction rate of 50–75% [84], and diabetes mellitus, where retrograde ejaculation may be present in a third of sufferers [85]. Retrograde ejaculation due to surgery or diabetes may respond to sympathomimetic medications in a third of cases [86], otherwise surgical sperm retrieval for ART is required. Sperm freezing may be also performed prior to cytotoxic therapies for nonmalignant conditions such as glomerulonephritis [87] and inflammatory bowel disease [88]. However, there is a paucity of data evaluating clinical outcomes following sperm freezing for these conditions.

## 7. Challenges to the Funding of Sperm Freezing

It is generally accepted that patients at high risk of infertility from cancer therapy should be offered sperm freezing. However, there is a paucity of data guiding how the patient and clinician should decide upon the duration of sperm freezing; clinical practice therefore varies and may depend on clinical requirements and funding. In the UK, the Human Fertilisation and Embryology Authority (HFEA) allows patients with persistent infertility to consent for sperm freezing for up to 55 years [89]. However, National Health Service (NHS) funding for sperm freezing may be limited to 5–10 years per patient, depending on the region of the UK. In other healthcare systems funded by private insurance or direct self-payment, referral for sperm freezing and the duration of sperm freezing may be driven by affordability, as with other forms of fertility therapy [90]. Disposal of sperm samples may be required by a sperm bank when fertility has recovered or funding for storage has expired. However, fear of litigation may limit the ability of sperm banks to dispose of samples if patients do not consent to this action [91,92].

## 8. Ethical Challenges in Sperm Freezing

Sperm freezing poses several ethical issues which must balance the needs of the donor, recipient and resulting offspring. Anonymity of sperm donation has been standard practice in many countries [93], but there is now an increasing trend for nonanonymous donation, whether voluntary or mandated by law [94]. For example, children conceived using sperm donated after April 2005 in the UK have the right to access the name, date of birth and last known address of their biological father when reaching the age of 18 years, but have no inheritance rights [95]. It has been feared that nonanonymous donation may reduce the pool of sperm donors, but some published data contradict this assumption [96]. Furthermore, it is important to consider that not all donors are against removing the anonymity for sperm donation [97,98], and current sperm donor recipients may be more willing to inform their children about the biological origin of their conception when compared with sperm donor recipients in the past [99,100].

The numbers of offspring sired from single donors is also a common ethical concern due to the risks of unintentional consanguinity. Guidelines from the Practice Committee of ASRM and Practice Committee of Society for Assisted Reproductive Technology recommend that sufficient records should be maintained to limit the utilization of sperm from single donors; in a population of 800,000 they recommended that no more than 25 live births result per donor [72]. In the UK, the HFEA recommend that sperm from a single donor cannot produce children for more than ten families [89]. Differing guidelines may reflect different population densities and therefore reflect differing risks of consanguinity.

It is routine practice that frozen sperm may be used posthumously by a named partner following the written permission of the patient. However, the question of whether posthumous retrieval and use of sperm by the surviving partner of a patient be permitted without written permission, remains a subject of debate. Sperm remain viable in situ up to 36 h following death [101], and can be retrieved by orchiectomy, removal of the epididymis, epididymal aspiration or testicular biopsy [102]. Countries such as Israel permit the retrieval and usage of sperm without written consent from the male partner by assuming these wishes are in place during marriage; conversely, such practice was absolutely prohibited other countries such as the UK [103]. However, a judge in the UK recently ruled that the widow of male survivor of cancer could use his sperm for fertility treatment for eight years after the expiry of his written permission, in order to allow sufficient time for grieving prior to any fertility therapy [104]. The Ethics Committee of the ASRM recommend that without written consent of the male partner, the request of a surviving spouse can be considered by referral to an ethics panel [103].

## 9. Future Avenues for Fertility Preservation

Mature sperm begin to appear in the ejaculate during puberty. Although sperm suitable for freezing can be collected in any boy with pubertal development, a recent study of 12–17 year old boys suggested that cryopreservation was most feasible in those older than 13 years [105]. Survival rates of childhood cancer are increasing [106]; it is therefore important to investigate whether fertility preservation is possible in prepubescent boys with cancer. Spermatogonial stem cells (SSCs) or testicular tissue may be cryopreserved by slow freezing using 1.5 M dimethylsulphoxide and 0.15 M sucrose as cryoprotectants [107]. Research investigating whether frozen SSC or testicular tissue samples can be used to restore fertility is ongoing [108]. SSCs can be successfully cultured in vitro in a supplemented human embryonic stem cell culture medium without losing their stem cell properties [109]. Spermatogenesis can be restored by injecting a SSC suspension into mice rendered infertile by busulfan therapy. One study demonstrated that of the 104 infertile mice receiving a SSC suspension injection to the testes, 38 (36.5%) regained normal levels of spermatogenesis [110]. Furthermore, spermatozoa are observed in the ejaculate of adult and prepubertal rhesus monkeys sterilized by alkylating chemotherapy followed by injection of SSCs into the rete testes [111]; these sperm can fertilize oocytes during intracytoplasmic sperm injection (ICSI). Collectively, these data suggest that SSC autotransplantation has future therapeutic potential for boys undergoing cancer therapy. However, some questions remain unanswered: will SSC autotransplantation confer a risk of reintroducing malignant cells? What is the epigenetic stability of SSCs is following cryopreservation [112]? Fluorescence-activated cell sorting (FACS) prior to SSC transplantation has been proposed as a way to mitigate the risk of reintroducing malignancies [113]. Autologous testicular tissue transplantation provides an alternative approach for treating infertile patients undergoing treatment for cancer prior to puberty. Testicular tissue transplantation has been shown to yield full spermatogenesis in prepubescent monkeys, but the rate of autograft survival was only 5% [114]. The rate of successful grafts using testicular tissue transplantation therefore appears lower than SSC injection. It is also important to consider the theoretical risk of auto-inoculating cancer cells back to the patient when autotransplanting testicular tissue, which must be mitigated with robust measures to ensure safety [108]. SSC and testicular tissue are therefore emerging technologies, which have the potential to extend the benefits of fertility preservation to prepubescent boys in the future. However, further clinical studies are required to establish their efficacy and safety before their routine use is recommended [108].

## 10. Conclusions

Sperm banking is an established and effective mode of preserving fertility in at-risk patients and for treating couples who would otherwise be unable to conceive. However, evidence suggests that access to fertility preservation services could be improved; closer collaboration between clinicians and sperm bank providers is required to ensure patients with cancer can benefit from referral in a timely fashion. It is also important to consider that widening access to sperm banking facilities is likely to have implications for the treatment costs in affected patients. While malignancy is the most common indication for fertility preservation, the large number of conditions, risk factors and treatments having the potential to impair sperm function requires all healthcare providers to enquire about the reproductive wish of their patients, with a view to minimize potential negative effects on fertility. Perhaps the biggest challenge relevant to sperm banking is the ever-expanding list of indications in which it has become incorporated. Regulations for sperm banking were formulated historically to treat men with cancer or heterosexual couples with infertility. The clinical landscape of sperm banking has already changed dramatically, which is illustrated by the increasing utilization of cryopreservation by homosexual and transgender couples. Furthermore, we cannot exclude that further developments such as genetically modifying gametes will become a reality in the future. Further research is needed to develop technologies for fertility preservation prior to puberty, to predict and perhaps prevent long-term gonadal toxicity following cancer therapy, and to refine established policies to deliver the greatest clinical impact for patients.

## Figures and Tables

**Table 1:** Risk factors for infertility in men with cancer. TMC—total motile count; IUI—intrauterine insemination; ESR—erythrocyte sedimentation rate; CED—cumulative effective dose; MOPP—Mustargen, Oncovin, Procarbazine, Prednisone; GvHD—graft versus host disease; RPLND—retroperitoneal lymph node dissection.

Risk Factor	Example	Notes
Malignancy	Testicular carcinoma	59.1% of patients at risk of TMC below threshold for IUI [31] Age and advanced cancer stage do not appear to increase sub-fertility risk [32]
	Leukemia (myeloid and lymphoid types)	89% reduction in TMC in myeloid, 60% in lymphoid Limited risk of TMC below threshold for use in IUI [23]
	Hodgkin’s lymphoma	Subfertile risk in widespread disease and with raised ESR [33] No statistically significant risk of TMC below threshold for use in IUI [31]
Chemotherapy	**High risk**	
	Cyclophosphamide	Impaired spermatogenesis unlikely when the CED less than 4000 mg/m^2^ [34]
	Procarbazine	Common with doses above 4 g/m^2^ [35]
	Ifosfamide	Two-thirds subfertile with dose >60 g/m^2^ [36]
	Chlormethine	90–100% of patients experience prolonged azoospermia when used in MOPP regime [37]
	Busulfan	Significant effects after single doses [38]
	Melphalan	Significant azoospermia after 2–3 months [39]
	Chlorambucil	Similar effect to mustine leading to decreased fertility [40]
	**Medium risk**	
	Cisplatin	<2 cycles does not adversely affect fertility [41]
	Carboplatin	Rates of azoo- and oligo-spermia may improve from 65% pretreatment to 42% post-treatment [42]
	Doxorubicin	Active sperm production within short timeframes expected [43]
	**Low risk**	
	Vincristine	Included within a number of regimes known to transient infertility in males [44]
	Methotrexate	Some case reports of reversible fertility [45]
	Dactinomycine	Number of studies suggest no detrimental effect on spermatogenesis [46,47]
	Bleomycin	One study showed relative risk for infertility of 1.55 [26]
	Mercaptopurine	Mouse studies showing adverse effects on sperm production [48], and documented case report of oligozoospermia [49]
	Vinblastine	Commonly used in gonadotoxic regimens and proven deleterious effect on mouse spermatozoa [50]
Radiation	Testicular malignancy	May confer no adverse effects on spermatogenesis, with a post-treatment paternity rate of 66% [51]
	Total body irradiation	Substantial recovery of spermatogenesis, particularly in patients aged <25 years, and if patients remain free of chronic GvHD [52]
	Prostate carcinoma	Damage shown to occur at doses ≥70 Gy [53]
	Rectal carcinoma	Effect seen at mean cumulative radiation exposure 3.56 Gy [54], reduced with high-dose-rate brachytherapy [55]
	Cranial radiotherapy	24 Gy given before age 10 results in 9% of the fertility of controls, not seen in treatment given in older age groups [56]
Biological therapeutics	Tyrosine kinase inhibitors e.g., imatinib	No randomized controlled trials currently to quantify risk
	mTOR inhibitors e.g., everolimus	No randomized controlled trials currently to quantify risk
Surgery	Orchiectomy	One year post orchiectomy and cisplatin-based chemotherapy normozoospermia seen in 64% After 3–5 years recovery found in 80% [57]
	Retroperitoneal lymph node dissection (RPLND)	Ejaculation preserved in significantly more patients undergoing nerve-sparing RPLND (89%) compared to modified bilateral template RPLND (11%) [58]
